# Exogenous Application of Alpha-Lipoic Acid Mitigates Salt-Induced Oxidative Damage in Sorghum Plants through Regulation Growth, Leaf Pigments, Ionic Homeostasis, Antioxidant Enzymes, and Expression of Salt Stress Responsive Genes

**DOI:** 10.3390/plants10112519

**Published:** 2021-11-19

**Authors:** Montaser H. M. Youssef, Aly Raafat, Ahmed Abou El-Yazied, Samy Selim, Ehab Azab, Ebtihal Khojah, Nihal El Nahhas, Mohamed F. M. Ibrahim

**Affiliations:** 1 Department of Agricultural Botany, Faculty of Agriculture, Ain Shams University, Cairo 11566, Egypt; montaserhassan@agr.asu.edu.eg (M.H.M.Y.); alyraafat@agr.asu.edu.eg (A.R.); 2 Department of Horticulture, Faculty of Agriculture, Ain Shams University, Cairo 11566, Egypt; ahmed_abdelhafez2@agr.asu.edu.eg; 3 Department of Clinical Laboratory Sciences, College of Applied Medical Sciences, Jouf University, Sakaka 2014, Saudi Arabia; sabdulsalam@ju.edu.sa; 4 Department of Food Science and Nutrition, College of Sciences, Taif University, P.O. Box 11099, Taif 21944, Saudi Arabia; e.azab@tu.edu.sa (E.A.); eykhojah@tu.edu.sa (E.K.); 5 Botany and Microbiology Department, Faculty of Science, Alexandria University, Alexandria 21515, Egypt; nihal.elnahhas@alexu.edu.eg

**Keywords:** *Sorghum bicolor* L. moench, α-lipoic acid, salinity, transporter proteins and oxidative stress

## Abstract

In plants, α-Lipoic acid (ALA) is considered a dithiol short-chain fatty acid with several strong antioxidative properties. To date, no data are conclusive regarding its effects as an exogenous application on salt stressed sorghum plants. In this study, we investigated the effect of 20 µM ALA as a foliar application on salt-stressed sorghum plants (0, 75 and 150 mM as NaCl). Under saline conditions, the applied-ALA significantly (*p* ≤ 0.05) stimulated plant growth, indicated by improving both fresh and dry shoot weights. A similar trend was observed in the photosynthetic pigments, including Chl a, Chl b and carotenoids. This improvement was associated with an obvious increase in the membrane stability index (MSI). At the same time, an obvious decrease in the salt induced oxidative damages was seen when the concentration of H_2_O_2_ and malondialdehyde (MDA) was reduced in the salt stressed leaf tissues. Generally, ALA-treated plants demonstrated higher antioxidant enzyme activity than in the ALA-untreated plants. A moderate level of salinity (75 mM) induced the highest activities of superoxide dismutase (SOD), guaiacol peroxidase (G-POX), and ascorbate peroxidase (APX). Meanwhile, the highest activity of catalase (CAT) was seen with 150 mM NaCl. Interestingly, applied-ALA led to a substantial decrease in the concentration of both Na and the Na/K ratio. In contrast, K and Ca exhibited a considerable increase in this respect. The role of ALA in the regulation of K^+^/Na^+^ selectivity under saline condition was confirmed through a molecular study (RT-PCR). It was found that ALA treatment downregulated the relative gene expression of plasma membrane (SOS1) and vacuolar (NHX1) Na^+^/H^+^ antiporters. In contrast, the high-affinity potassium transporter protein (HKT1) was upregulated.

## 1. Introduction

Salinity is considered one of the most compelling environmental challenges encountered worldwide by the agricultural sector [1]. Generally, salt stress can cause significant damage to biodiversity, ecosystems, human health, and natural resources [2]. Nowadays, this problem has been exacerbated in several regions of the world due to the adverse impacts of human activities, frequent climate changes, scarcity of freshwater, and a limitation of arable lands [1,3]. Recently, salt affected lands worldwide were estimated to be 1125 million hectares [4]. In the next few years, these areas are expected to increase with the exponential growth of the global population, threatening food security [1,4,5]. Therefore, achieving an increase in agricultural food production under saline conditions has become a critical area of concern.

Under salt stress conditions, both the behavior of plants and their interaction with the stressful factor have been found to be extremely complex, leading to changes at the morphological, physiological, biochemical, and molecular levels [6,7]. This complexity can ultimately trigger varying degrees of stress adaptation among the salt-tolerant and sensitive plant species [8,9]. In this context, plants are developing diverse defense strategies and mechanisms to reduce the detrimental effects and toxicity of salt ions affecting different developmental stages and metabolic pathways. These processes contain multiple steps. First, stimulation of the antioxidative systems (enzymatic and non-enzymatic) is necessary to keep the reactive oxygen species (ROS) under control [6,7]. In general, maintaining these cytotoxic molecules (ROS) at low levels can allow them to act as beneficial, significant signaling molecules involved in different metabolic events [6,7,10,11,12]. Secondly, maintaining a low Na^+^/K^+^ ratio in tissues is a common response in a wide array of plants. This response can occur when the gene expression of a number of high affinity Na^+^ and/or K^+^ transporter proteins, such as SOS1, HKT1 and NHX1, is altered [6,13]; Furthermore, regulating the expression of these genes is usually concomitant with maintaining the cell membranes stability index, photosynthetic pigments, and enhancing plant growth and development [6,7,8].

Sorghum (*Sorghum bicolor* L. Moench; family, Poaceae) is the 5th most cultivated cereal crop in arid and semiarid regions worldwide [14]. It is extremely economically important due to multiple uses in human nutrition [15] and as a fodder for animals [16,17]. Despite sorghum being a C4 plant, which is generally considered a tolerant plant to diverse stressful factors including drought, salinity, and high temperatures [18,19], its growth and productivity can be significantly affected under severe adverse conditions in particular salinities [8,17]. Thus, greater attention is needed to find an optimal solution to these challenges.

In this study, α-lipoic acid (1, 2-dithiolane-3-pentanoic acid; ALA) was investigated, as it is one of the most promising and effective solutions that can reduce the detrimental effects of salinity stress on sorghum plants. It can maintain its antioxidative power and protective impacts against diverse stresses in both its reduced and oxidised form [20]. Its antioxidant capacity depends on two sulfhydryl moieties [21] which enable it to scavenge free radicals and chelate metals [22]. Under salt stress conditions, ALA was reported to mitigate oxidative damage and enhance growth and root formation of canola seedlings [23]. Moreover, exogenous ALA has been suggested to improve photosynthesis and induce tolerance mechanisms of several plant species under diverse environmental stresses [20,24,25].

This study was conducted to determine the role of ALA as an exogenous application and its possible ameliorative effects in sorghum plants grown under saline conditions. These effects were examined through several aspects: strengthening the antioxidant capacity, modifying the ionic homeostasis, maintaining cell membrane stability, and stimulating the growth of stressed plants.

## 2. Materials and Method

### 2.1. Growth Conditions and Experimental Design

A pot experiment was conducted from 16 May 2021 to 14 July 2021 at the experimental farm located in the Department of Agricultural Botany, Faculty of Agriculture, Ain Shams University, Cairo, Egypt. The seeds of grain sorghum (*Sorghum bicolor* L. Moench; CV. Dorado) were provided by the Agriculture Research Center, Egypt. Sodium hypochlorite 0.5% was used to sterilize the surface of seeds for 5 min, after which they were washed with distilled water several times. Seeds were sown in black plastic pots (30 cm diameter) filled with 16 kg pre-washed sand. After 2 weeks, the pots containing seedlings homogenous in size and form (two seedlings/pot) were then regularly irrigated with half strength Hoagland’s solution modified by adding 0, 75 or 150 mM NaCl every 2 days. Irrigation was performed three times a week with half strength Hoagland’s solution (two times with NaCl and the last time without NaCl to prevent average soil salinity from rising above the studied levels with time (leaching requirement)). The total volume of solution ranged between 0.8–1.1 L/pot every irrigation, adjusting with increased growth of the plants and the rate of evapotranspiration (ET) (the volume was calculated according to the reduction in water holding capacity by weight method). Under each level of salinity, pots were divided to two subgroups to apply ALA (0 or 20 µM) as foliar application. To determine the concentration of ALA in this study, a quick preliminary experiment was conducted for 25 days with different concentrations (0, 5, 10, 20, 50 and 100 µM) based on the chlorophyll SPAD readings using a digital chlorophyll meter (Minolta SPAD-502, Marunouchi, Japan).

Each pot of ALA-untreated plants was sprayed every 10 days with 15 mL of a solution containing distilled water and 0.05% (*v*/*v*) Tween-20 (non-ionic surfactant), whereas each pot of ALA-treated plants was sprayed by 15 mL of a solution containing 20 µM ALA plus 0.05% (*v*/*v*) Tween-20. All foliar treatments were stopped 15 days before the date of sampling (60 days after sowing), at which point leaves were collected to measure and analyze the different parameters. The experimental layout was a complete randomized design (CRD) with three replicates.

### 2.2. Determination of Growth Parameters

Shoot fresh weight was immediately estimated after sampling using digital balance, whereas shoot dry weight was determined by drying the samples in an air-forced ventilated oven at 105 °C.

### 2.3. Membrane Stability Index (MSI), Hydrogen Peroxide and Lipid Peroxidation

Cell membrane stability was measured by the electrolyte leakage technique as described by Singh, et al. [26] with some modifications [27]. Samples from each treatment were selected randomly from fully expanded leaves. Ten leaf discs (1.8 cm diameter) were cut, cleaned well and incubated in 10 mL deionized water for 24 h on a shaker. After that EC_1_ values of contents were measured by EC meters (DOH-SD1, TC-OMEGA, Stamford, CT, USA). Then, samples were autoclaved at 120 °C for 20 min to determine the values of EC_2_. Cell membrane stability index was calculated using the following equation: MSI = 1 − (EC_1_/EC_2_) × 100.

Hydrogen peroxide (H_2_O_2_) concentration was determined according to [28] with some modifications. Leaf samples of 0.5 g were homogenized in 3 mL of 1% (*w/v*) trichloroacetic acid (TCA). The homogenate was centrifuged at 10,000 rpm and 4 °C for 10 min. Subsequently, 0.75 mL of the supernatant was added to 0.75 mL of 10 mM K-phosphate buffer (pH 7.0) and 1.5 mL of 1 M KI. H_2_O_2_ concentration was evaluated by comparing its absorbance at 390 nm to a standard calibration curve. The concentration of H_2_O_2_ was calculated from a standard curve plotted in the range from 0 to 15 nmol mL^−1^.

The level of lipid peroxidation was measured by the determination of malondialdehyde (MDA) as described by Heath and Packer [29]. Frozen tissues were homogenized in 0.1% (*w/v*) trichloroacetic acid (TCA). The extraction ratio was 10 mL for each gram of plant tissues. The homogenate was centrifuged at 4500 rpm for 15 min. The reaction mixture contained 1 mL from the supernatant and 4 mL 0.5% (*w/v*) thiobarbituric acid (TBA) dissolved in 20% (*w/v*) TCA. The mixture was heated in boiling water for 30 min then the mixture was cooled at room temperature and centrifuged at 4500 rpm for 15 min. The absorbance of the supernatant was measured at 535 nm and corrected for non-specific turbidity at 600 nm using a spectrophotometer (UV-1601PC; Shimadzu, Tokyo, Japan). The MDA concentration (nmol.g^−1^ FW) was calculated using Δ OD (A532–A600) and the extinction coefficient (ε = 155 mM^−1^cm^−1^).

### 2.4. Determination of Leaf Pigments

Chlorophyll a, b and total chlorophyll was determined as described by Costache, et al. [30] with some modification, small pieces of fresh leaves (0.5 g) was submerged into 10 mL pure acetone for 24 h/4 °C. The absorbance was measured at 645 and 663 nm respectively. The concentration was calculated using the following equations:

Chlorophyll a (mg/g FW) = 11.75 A662 − 2.350 A645 × (V/1000 × W)

Chlorophyll b (mg/g FW) = 18.61 A645 − 3.960 A662 × (V/1000 × W)

where, A is the absorbance at 645 and 663 nm, V is the Final volume of chlorophyll extract in pure acetone and W is the fresh weight of tissue extract.

Carotenoids were quantified using the acetone and petroleum ether method as described by de Carvalho, et al. [31] using the following formula:

Carotenoids (mg/g FW) = A_450_ × V (mL) × 10/ [A^1%^_1cm_ × W (g)]

where A_450_ = Absorbance at 450 nm, V = Total extract volume; W = sample weight; A^1%^_1cm_ = 2592 (β-carotene coefficient in petroleum ether).

### 2.5. Assay of Antioxidant Enzymes

To prepare the extraction of enzyme and soluble proteins, 0.5 g fresh leaves was homogenized in 4 mL 0.1 M sodium phosphate buffer (pH 7.0) containing 1% (*w:v*) polyvinylpyrrolidon (PVP) and 0.1 mM EDTA, centrifuged at 10,000× *g* for 20 min at 4 °C and then the supernatant was used for assays. To calculate the specific activity of enzymes, the concentration of total soluble protein was evaluated by the method of Bradford [32]. All studied enzyme activities were measured using a spectrophotometer (UV-1601PC; Shimadzu, Tokyo, Japan) as following:

Superoxide dismutase (SOD) (EC 1.15.1.1) assay was based on the method described by Beyer and Fridovich [33]. The reaction mixture with a total volume of 3 mL contained 100 μL total soluble protein extract, 50 mM phosphate buffer (pH 7.8), 75 μM NBT, 13 mM L-methionine, 0.1 mM EDTA and 0.5 mM riboflavin. The reaction was initiated by the addition of riboflavin then the reaction mixture was illuminated for 20 min with 20 W flourscent lamp. One unit of enzyme activity was defined as the amount of enzyme required to result in a 50% inhibition in the rate of nitro blue tetrazolium (NBT) reduction at 560 nm.

Catalase (CAT) (EC 1.11.1.6) activity was measured by monitoring the decrease in absorbance at 240 nm as described by Cakmak, et al. [34]. The reaction mixture with a total volume of 3 mL contained 15 mM H_2_O_2_ in 50 mM phosphate buffer (pH = 7). The reaction was initiated by adding 50 μL total soluble protein extract. The activity was calculated from extinction coefficient (ε = 40 mM^−1^ cm^−1^) for H_2_O_2_. One unit of enzyme activity was defined as the decomposition of 1 μmol of H_2_O_2_ per minute.

Guaiacol peroxidase (G-POX) (EC1.11.1.7) activity was quantified by the method of Dias and Costa [35] with some minor modifications. The assay mixture (100 mL) contained 10 mL of 1% (*v*/*v*) guaiacol, 10 mL of 0.3% H_2_O_2_ and 80 mL of 50 mM phosphate buffer (pH = 6.6). The volume of 100 µL of the total soluble protein extract was added to 2.9 mL of the assay mixture to start the reaction. The absorbance was recorded every 30 s for 3 min at 470 nm. One unit of G-POX was defined as the amount of enzyme that caused the formation of 1 µM of guaiacol dehydrogenation per minute (the final product of the oxidized guaiacol by H_2_O_2_).

The activity of ascorbate peroxidase (APX) (EC 1.11.1.11) was determined according to Nakano and Asada [36]. The decrease of absorbance at 290 nm was monitored for 3 min. The reaction mixture with a total volume of 3 mL included 100 µL total soluble protein extract, 50 mM phosphate buffer (pH 7), 0.1 mM EDTA, 0.5 mM ascorbic acid, and 0.1 mM H_2_O_2_. The reaction was initiated by the addition of H_2_O_2_. One unit of enzyme activity was defined as the amount of enzyme required for oxidation of 1 µmol of ascorbate per minute. The rate of ascorbate oxidation was calculated using the extinction coefficient (ε = 2.8 mm^−1^ cm^−1^). 2.6. Determination of Na, K and Ca

Leaf mineral concentrations of Na, K and Ca were determined using the flame photometric method (Jenway, Staffordshire, UK) as described by Havre [37].

### 2.6. Gene Expression

Total mRNA was isolated from 0.5 g sorghum leaves under salinity stress levels (0, 75, 150 mM NaCl) and 20 µM α Lipoic acid as foliar application treatment compared with the untreated plant (control experiment). Total RNA is easily purified from plant leaves tissues by RNeasy Tissue Kits (Qiagen, Maryland, USA) according to the manufacturer’s protocol. Quantification and quality of the purified RNA was checked with a NanoDrop spectrostar (BMG LABTECH, Saitama, Japan), and analyzed on 1% agarose gel. For each sample, Total RNA (5 μg) was reverse transcribed to complementary cDNA in a reaction mixture consists of 2.5 μL 2.5 mM dNTPs, 2.5 μL MgCl_2_, 1.0 μL oligo dT primer (10 pml/μL), 2.5 μL 5X buffer, 0.2 μL (5 Unit/μL) reverse transcriptase (Promega, Baden-Württemberg, Germany), RT-PCR amplification was performed in a thermal cycler PCR, at 42 °C for 1.5 h and 80 °C for 20 min. Quantitative Real time PCR carried out on 1 μL diluted cDNA by triplicate using the real time analysis using (Rotor-Gene 6000, Qiagen, Hilden, Germany) system and the primer sequences used in qRT-PCR were given in Table 1. Primers of Salt Overly Sensitive (SOS), high-affinity potassium transporter 1 (HKT1) and the members of tonoplast-localized Na^+^/H^+^ antiporter (NHX) genes and GAPDH housekeeping gene (reference gene) were used for gene expression analysis used a SYBR^®^ Green based method. A total reaction volume of 20 µL was used. Reactions mixture consists of 2 µL of template, 10 µL of SYBR Green Master Mix, 2 µL of reverse primer, 2 µL of forward primer, and sterile dist. water for a total volume of 20 µL. PCR assays were performed using the following conditions: 95 °C for 15 min followed by 40 cycles of 95 °C for 30 s and 60 °C for 30 s. The CT of each sample was used to calculate ΔCT values (target gene CT subtracted from β-Actin gene CT). The relative gene expression was determined using the 2^−ΔΔCt^ method [38].

### 2.7. Statistics

One way ANOVA procedure was followed using SAS [39] software. Means ± SD were calculated from three replicates and Tukey’s multiple range test (*p* ≤ 0.05) was used to determine significant differences between means.

## 3. Results

### 3.1. Effect of ALA on Growth Parameters

**Figure 1:**
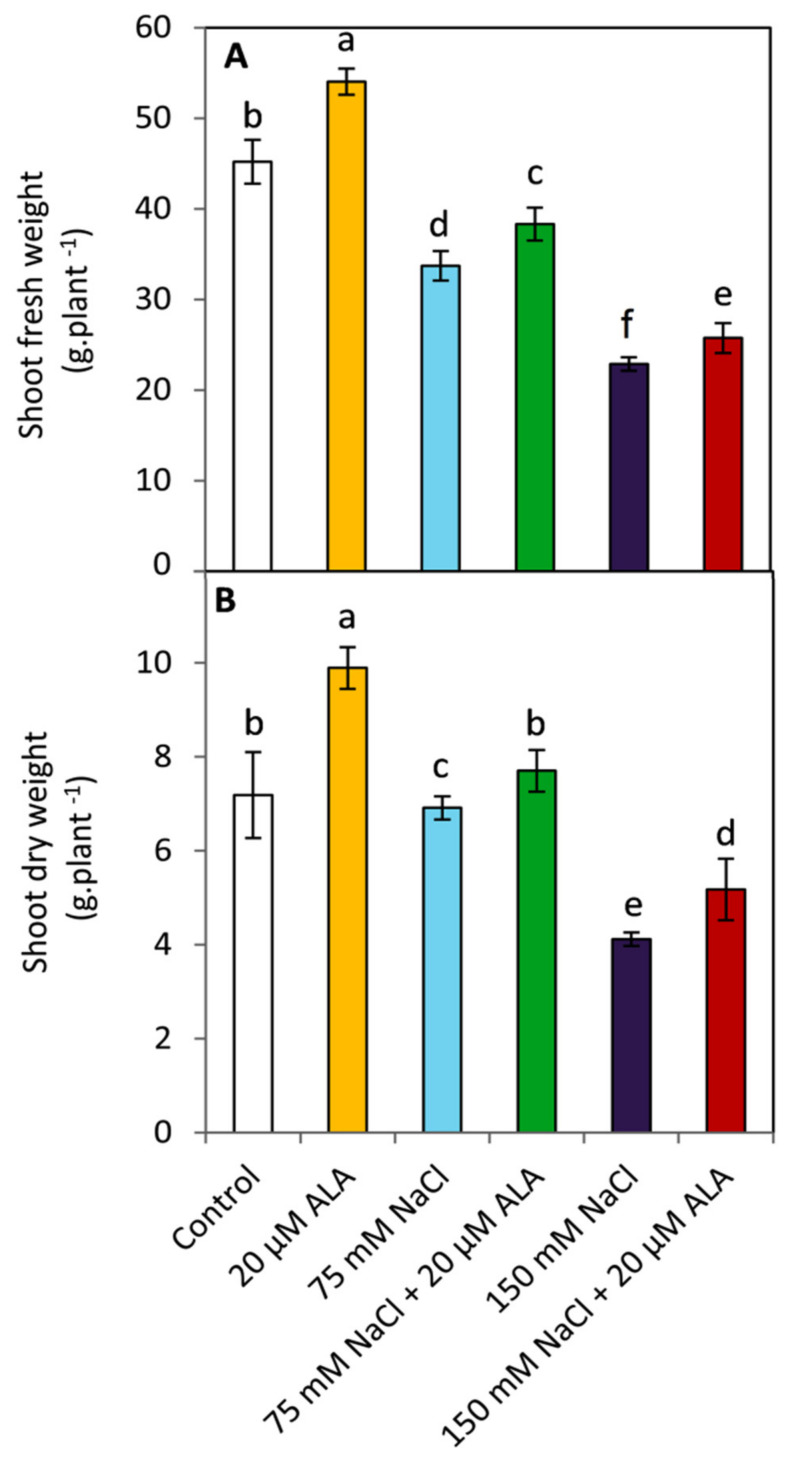
Effect of salinity stress as NaCl (0, 75 and 150 mM) and the foliar application by α-lipoic acid (ALA; 0 and 20 µM) on (**A**) shoot fresh and (**B**) dry weight of sorghum plants. For each parameter, the mean values ± SD followed by a different letter are significantly (*p* ≤ 0.05) different according to Tukey’s range test.

Exposing sorghum plants to salt stress significantly (*p* ≤ 0.05) reduced the growth parameters compared to the unstressed plants (Figure 1). In general, the treatment with the lowest values in plant height, fresh weight, dry weight, and leaf area was the higher NaCl concentration (150 mM). In contrast, ALA-treated plants showed a significant (*p* ≤ 0.05) improvement in all studied growth parameters under non-saline conditions. A similar trend was observed in respect to plant height, fresh weight, and dry weight in ALA treated plants under saline conditions (75 and 150 mM NaCl). However, this effect was not significant (*p* ≤ 0.05) in leaf area under both examined levels of salinity.

### 3.2. Effect of ALA on the Membranes’ Stability and Leaf Oxidative Damage

**Figure 2:**
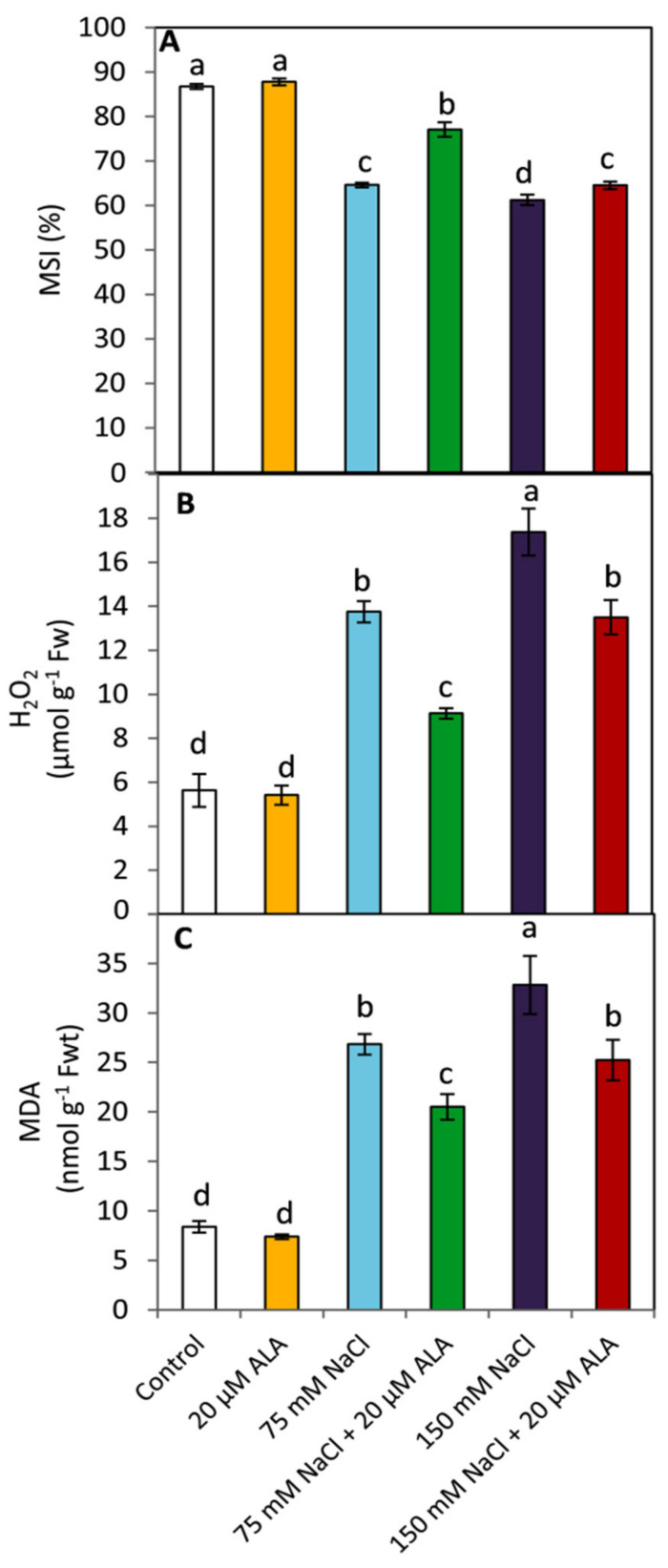
Effect of salinity stress as NaCl (0, 75 and 150 mM) and the foliar application by α-lipoic acid (ALA; 0 and 20 µM) on (**A**) membranes stability index (MSI), (**B**) H_2_O_2_ and (**C**) malondialdehyde (MDA) of sorghum plants. For each parameter, the mean values ± SD followed by a different letter are significantly (*p* ≤ 0.05) different according to Tukey’s range test.

Exposing sorghum plants to salt stress significantly (*p* ≤ 0.05) decreased the membrane stability index (MSI) in parallel with raising NaCl concentrations, up to 150 mM, compared to the unstressed plants (Figure 2A). This decrease was associated with a significant (*p* ≤ 0.05) increase in the leaf oxidative damage and the rate of lipid peroxidation as indicated by the elevated the concentration of H_2_O_2_ and MDA, respectively (Figure 2B,C). Applied-ALA was shown to significantly enhance (*p* ≤ 0.05) MSI under both investigated levels of salinity. Simultaneously, this effect was concomitant with a significant (*p* ≤ 0.05) decrease in the concentration of H_2_O_2_ and MDA.

### 3.3. Effect of ALA on the Photosynthetic Pigments

**Figure 3:**
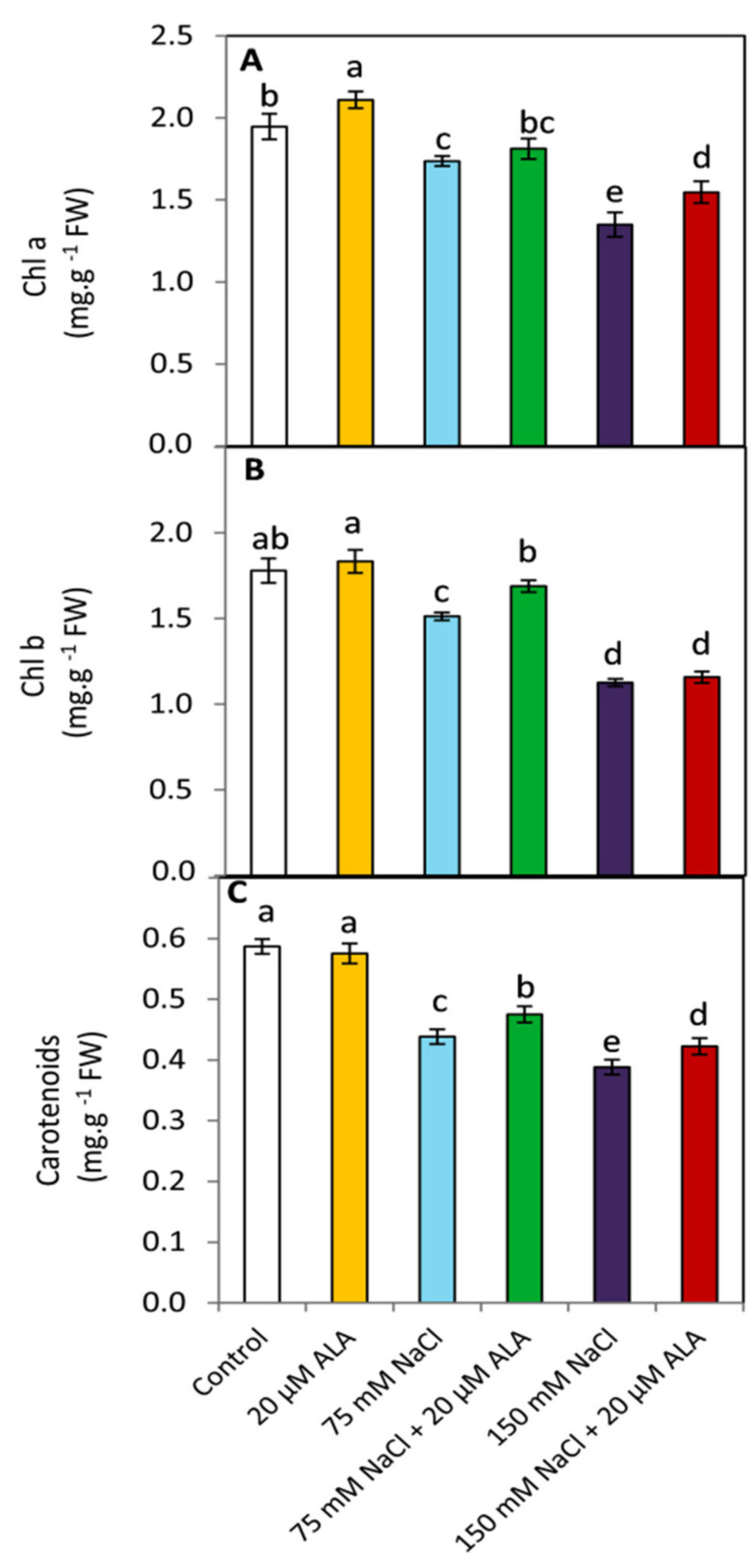
Effect of salinity stress as NaCl (0, 75 and 150 mM) and the foliar application by α-lipoic acid (ALA; 0 and 20 µM) on the leaf photosynthetic pigments of sorghum plants including (**A**) chlorophyll a, (**B**) chlorophyll b and (**C**) carotenoids. For each parameter, the mean values ± SD followed by a different letter are significantly (*p* ≤ 0.05) different according to Tukey’s range test.

Data illustrated in Figure 3 show that Chl a was positively and significantly (*p* ≤ 0.05) affected by the treatment of ALA under non-saline conditions; whereas, Chl b and carotenoids did not reveal any significant changes in this respect. Under saline conditions, Chl a, Chl b and carotenoids were significantly (*p* ≤ 0.05) decreased compared to the non-stressed plants. This negative effect was more destructive to all studied leaf pigments with increasing the level of salt stress. Otherwise, the treatment of ALA improved significantly (*p* ≤ 0.05) Chl b and carotenoids under the lower level of salinity. When plants were subjected to the higher level of salinity, ALA treatment led to maintaining significantly (*p* ≤ 0.05) the content of Chl a and carotenoids. These findings may indicate the protective effect of ALA on the photosynthetic machinery.

### 3.4. Effect of ALA on the Activities of Antioxidant Enzymes

**Figure 4:**
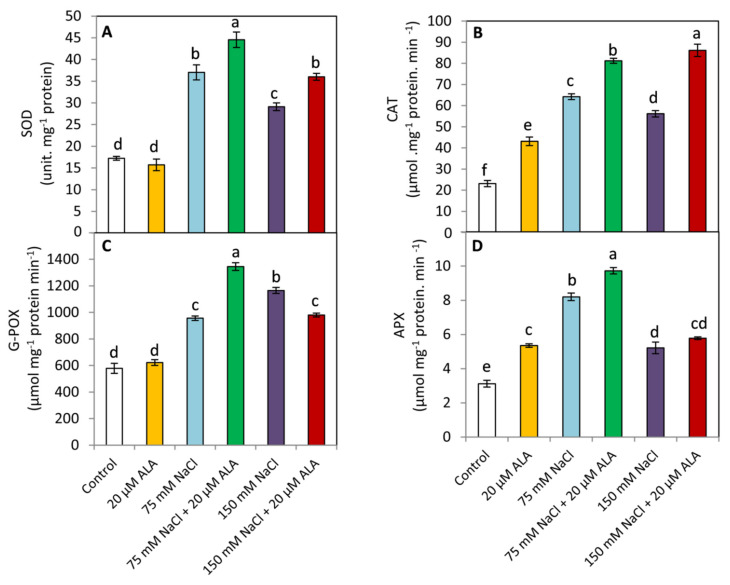
Effect of salinity stress as NaCl (0, 75 and 150 mM) and the foliar application by α-lipoic acid (ALA; 0 and 20 µM) on the activities of antioxidant enzymes including (**A**) superoxid dismutase (SOD), (**B**) catalase (CAT), (**C**) guaiacol peroxidase (G- POX) and (**D**) ascorpate peroxidase (APX) of sorghum plants. For each parameter, the mean values ± SD followed by a different letter are significantly (*p* ≤ 0.05) different according to Tukey’s range test.

Under saline conditions, the general tendency was that the activities of antioxidant enzymes including SOD, CAT, POX, and APX revealed a significant (*p* ≤ 0.05) increase compared to the unstressed plants (Figure 4). Under non saline conditions, applied-ALA significantly (*p* ≤ 0.05) increased the activities of CAT and APX; whereas, this effect did not reach the level of significance in respect to SOD and POX. On the other hand, ALA-treated plants showed a significant (*p* ≤ 0.05) increase in SOD, CAT compared to ALA-untreated plants under both investigated levels of salinity. This response was explicit in POX and APX under slight saline condition (75 mM), whereas, POX was significantly (*p* ≤ 0.05) decreased in the ALA-treated plants under the higher level of salinity (150 mM).

### 3.5. Effect of ALA on Na, K, Ca and Na/K Ratio

**Figure 5:**
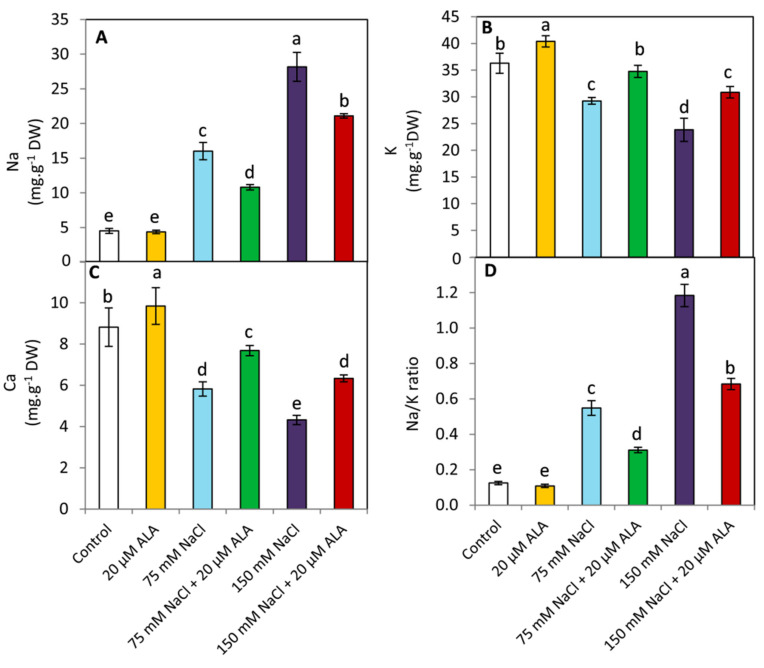
Effect of salinity stress as NaCl (0, 75 and 150 mM) and the foliar application by α-lipoic acid (ALA; 0 and 20 µM) on the leaf content of Na (**A**), K(**B**), Ca (**C**) and Na/K ratio (**D**) of sorghum plants. For each parameter, the mean values ± SD followed by a different letter are significantly (*p* ≤ 0.05) different according to Tukey’s range test.

Under saline conditions, the Na and Na/K ratio was significantly (*p* ≤ 0.05) increased in leaf tissues compared to the unstressed plants. In contrast, a significant decrease in the concentration of K and Ca was observed with raising the level of salinity (Figure 5). On the other hand, applied ALA achieved significant decrease (*p* ≤ 0.05) in the concentration of the Na and Na/K ratio under both examined levels of salinity. These influences were associated with an obvious and significant (*p* ≤ 0.05) enhancement of the concentration of K and Ca. These results imply that exogenous ALA may induce tolerance to salinity stress in sorghum plants by affecting the homeostasis of relevant salt stress ions.

### 3.6. Effect of ALA on the Expression of SOS1, NHX1 and HKT1

**Figure 6:**
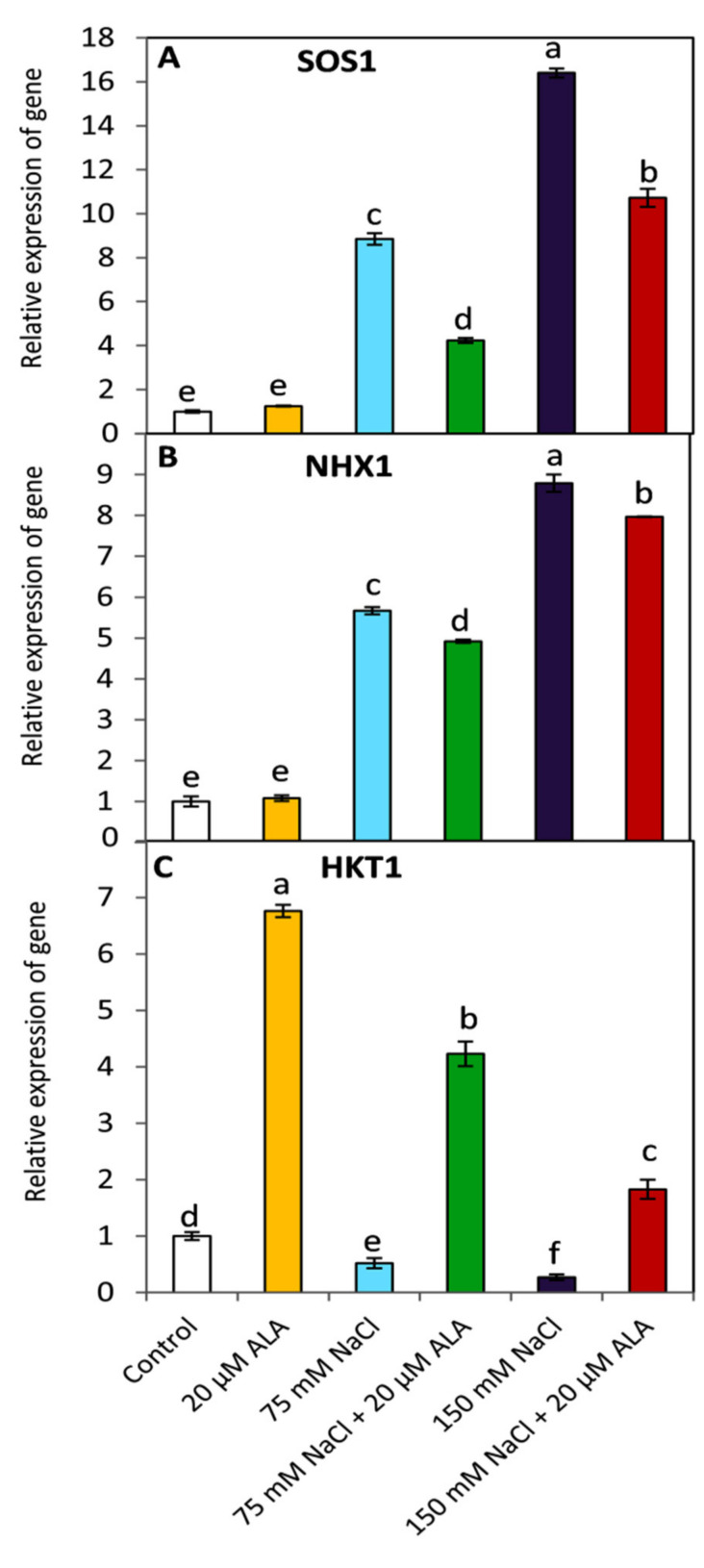
Effect of salinity stress as NaCl (0, 75 and 150 mM) and the foliar application by α-lipoic acid (ALA; 0 and 20 µM) on the relative expression of SOS1 (**A**), NHX1 (**B**) and HKT1 (**C**) of sorghum plants. For each parameter, the mean values ± SD followed by a different letter are significantly (*p* ≤ 0.05) different according to Tukey’s range test.

The relative expression of salt stress relevant genes (SOS1, NHX1 and HKT1) was investigated in this study (Figure 6). The results indicated that SOS1 and NHX1 were significantly (*p* ≤ 0.05) upregulated with increasing the level of salinity compared to those of non-saline condition. Conversely, an obvious and significant (*p* ≤ 0.05) downregulation in HKT1 was observed under both investigated levels of salinity stress (75 and 150 mM). On the other hand, applied-ALA led to a significant *p* ≤ 0.05) inhibition in the relative expression of SOS1and NHX1 compared to the ALA-untreated plants under the same level of salinity. On the contrary, ALA caused a significant (*p* ≤ 0.05) increase in the relative expression of HKT1 regardless the presence of salinity stress.

## 4. Discussion

Several studies have shown that salt stress can affect multiple morphological, physiological, biochemical, and molecular aspects of plants [6,17,40]. In this study, we observed that exposing plants to salt stress inhibited plant growth parameters, including plant height, fresh weight, dry weight, and leaf area as compared to the unstressed plants (Figure 1). This decrease in plant growth can be attributed to the modulation of cell cycle progression as well as inhibition of the rate of cell division [41]. Furthermore, elevating the level of salinity is a key factor in increasing osmotic stress and decreasing plant growth by affecting the ability of plants to uptake water [42,43]. In contrast, ALA-treated plants showed considerable enhancement in all of the studied growth parameters regardless of the presence of salinity stress. Alpha-lipoic acid is a potent antioxidant that is soluble in both water and lipids [44]. A previous report showed that ALA can enhance growth and root formation of the salt-stressed canola seedlings [23]. This stimulation may be due to enhanced photosynthesis and carbon fixation [24].

In this study, exposing plants to salt stress resulted in a significant decrease in their membrane stability index (MSI) and a greater increase in H_2_O_2_ and malondialdehyde (MDA) (Figure 2). Generally, under abiotic stress conditions, the excessive generation of reactive oxygen species (ROS) is a common response in many plant species to oxidative damage [45,46,47,48]. These harmful molecules can lead to destruction of cell membrane structure by affecting the structure and function of the protein and lipid bilayers [49]. In contrast, ALA-treated plants showed a significant enhancement in their MSI, as well as a parallel decrease in H_2_O_2_ and MDA. It has been found that exogenous ALA is a potent dithiol antioxidant and can mitigate oxidative damage by scavenging ROS that are produced under diverse environmental stresses, such as high salinity [23], drought [24], heavy metals [21,25], and osmotic stress [20].

Interestingly, the positive influence of ALA on maintaining the cell membrane stability index and reducing oxidative damage was positively reflected in the leaf content represented by the photosynthetic pigments i.e., Chl a, Chl b and carotenoids (Figure 3). Furthermore, it was observed that ALA treatment was more effective on improving the content of Chl a than it was on Chl b when treated with the higher level of salinity. This effect implies that under high levels of salinity, ALA as a potent antioxidant has a key protective role on Chl a (considered the major cofactor in the photochemical reactions inside chloroplast) [50]. It is well known that ABA (the major stress hormone in higher order plants under osmotic stress) is synthesized from a carotenoid intermediate [51,52]. In this study, under saline conditions, carotenoids were increased by ALA treatment. This effect could be attributed to improved cell membrane stability and water potential, affecting the biosynthesis of ABA and consequently maintaining the carotenoids content.

Under abiotic stress conditions, increasing the activity of antioxidant enzymes is necessary for ROS elimination [9,45,53,54]. In this study, the activities of SOD, CAT, POX, and APX were significantly increased under saline conditions (Figure 4). These responses occurred to restrict the excessive accumulation of superoxide radicals and H_2_O_2_ [55,56]. Under a low level of salinity (75 mM NaCl), ALA treatment caused a significant increase in the activity of SOD, CAT, POX, and APX. Several previous studies have suggested that ALA is able to induce the antioxidant systems (enzymatic and non-enzymatic) in plants under diverse abiotic stressors [20,23,25,44]. This effect may be attributed to the essential role of ALA as a part of several multi-enzyme complexes [57]. Under the highest level of salinity (150 mM NaCl), ALA-treated plants displayed a significant decrease in POX, while, SOD and CAT showed an opposite trend. No significant changes were detected in APX. These results imply that under severe levels of salinity stress, SOD and CAT are the two major antioxidant enzymes for scavenging superoxide radicals and H_2_O_2,_ respectively, in sorghum plants. In contrast, the decrease in the activity of POX in ALA-treated plants under high levels of salinity (150 mM NaCl) could be attributed to the antioxidative properties of ALA which relatively compensate for the role of POX in the ALA-untreated plants.

In plants, Na^+^ exclusion and reducing Na/K ratio in the sensitive tissue of the leaf are critical techniques for plant tolerance to saline stress. This response can be attributed to minimizing the toxic effect of Na^+^ on several cytosolic enzymes [58]. In the present study, NaCl-stressed plants demonstrated different compartmentalization of Na, K, and Ca in their leaf tissues (Figure 5). The increase in NaCl concentration was associated with a decrease in the concentration of K and Ca, making the reduction of the Na/K ratio very clear. These results were consistent with those obtained in several previous studies on many plant species [6,8,40,42]. On the other hand, ALA-treated plants revealed a significant decrease in the concentration of Na and the Na/K ratio in the leaf under both examined salinity levels. These influences were concomitant with greater improvement in the concentration of K and Ca. These effects could be attributed to the positive effect of ALA on the membrane stability index (Figure 2), which can affect plant water relation and the ability of plants to uptake K and Ca with transpiration stream. In this respect, similar results were reported in salt-stressed wheat seedlings [59].

To further understand the effect of ALA on Na and K, and subsequently the Na/K ratio, under saline conditions, using RT-PCR, the relative gene expression of some membrane transport proteins mediating Na and K transport was studied in this investigation. We found that the relative expression of SOS1 (plasma membrane Na^+^/H^+^ antiporter) and NHX1 (vacuolar Na^+^/H^+^ antiporter) were significantly downregulated in the ALA-treated plants under saline conditions (Figure 6A,B). These responses may enable plants to survive under saline conditions by excluding Na^+^ from the cytosol to the apoplast or the vacuole. In contrast, the high-affinity potassium transporter (HKT1) was upregulated in the ALA-treated plants compared to the untreated ones (Figure 6C), indicating that ALA enhanced K^+^/Na^+^ selectivity and thus the plant’s tolerance to salinity stress. These findings were previously discussed in this study (Figure 5).

## 5. Conclusions

From the result of this study, we can conclude that ALA can induce tolerance to salinity stress in sorghum plants. Data show that ALA has many protective aspects against salt stress through enhancing plant growth, the membrane stability index (MSI), and reducing oxidative damage. These responses were associated with increasing the activities of antioxidant enzymes (SOD, CAT, POX, and APX). Furthermore, ALA affected ionic homeostasis by reducing the uptake of Na and increasing K and Ca. This effect led to maintaining a lower Na/K ratio in leaf tissues. The explanation for these important influences is that exogenous ALA leads to a significant downregulation in the relative gene expression of plasma membrane (SOS1) and vacuolar (NHX1) Na^+^/H^+^ antiporters. At the same time, we show a considerable upregulation in the high-affinity potassium transporter protein (HKT1).

## Figures and Tables

**Table 1:** Oligonucleaotides primer pairs used for quantitative RT-PCR analysis.

Gene Name		Sequence	NCBI Accession
SOS1	F	5′-ACTTGCAGGAGGAATACAAC-3′	NM001176582
	R	5′- CGAGAAGAGAAGACCACATC-3′,	
HKT1	F	5′-TGCTAATGTTTATCGTGCTG-3′	HQ845286
	R	5′-AGGCTGATCCTCTTCCTAAC-3′	
NHX1	F	5′-CGTGATGTCGCATTACACCT-3′	AY270040
	R	5′- CTGGCAAACTCCCACTTCTC-3′	
GAPDH	F	5′-TGACGACATCAAGAAGGTGGTG-3′	NM_001082253
	R	5′-:GAAGGTGGAGGAGTGGGTGTC-3′	

## Data Availability

Data is contained within the article.

## References

[1] Tomaz A., Palma P., Alvarenga P., Gonçalves M.C. (2020). Soil salinity risk in a climate change scenario and its effect on crop yield. Climate Change and Soil Interactions.

[2] Payen S., Basset-Mens C., Núñez M., Follain S., Grünberger O., Marlet S., Perret S., Roux P. (2016). Salinisation impacts in life cycle assessment: A review of challenges and options towards their consistent integration. Int. J. Life Cycle Assess..

[3] Iglesias M.C.-A. (2020). A review of recent advances and future challenges in freshwater salinization. Limnetica.

[4] Hossain M.S. (2019). Present scenario of global salt affected soils, its management and importance of salinity research. Int. Res. J. Biol. Sci..

[5] Butcher K., Wick A.F., DeSutter T., Chatterjee A., Harmon J. (2016). Soil salinity: A threat to global food security. Agron. J..

[6] Alnusairi G.S., Mazrou Y.S., Qari S.H., Elkelish A.A., Soliman M.H., Eweis M., Abdelaal K., El-Samad G.A., Ibrahim M.F., ElNahhas N. (2021). Exogenous Nitric Oxide Reinforces Photosynthetic Efficiency, Osmolyte, Mineral Uptake, Antioxidant, Expression of Stress-Responsive Genes and Ameliorates the Effects of Salinity Stress in Wheat. Plants.

[7] El Nahhas N., AlKahtani M.D., Abdelaal K.A., Al Husnain L., AlGwaiz H.I., Hafez Y.M., Attia K.A., El-Esawi M.A., Ibrahim M.F., Elkelish A. (2021). Biochar and jasmonic acid application attenuates antioxidative systems and improves growth, physiology, nutrient uptake and productivity of faba bean (*Vicia faba* L.) irrigated with saline water. Plant Physiol. Biochem..

[8] Calone R., Sanoubar R., Lambertini C., Speranza M., Vittori Antisari L., Vianello G., Barbanti L. (2020). Salt tolerance and Na allocation in *Sorghum bicolor* under variable soil and water salinity. Plants.

[9] Abd Elhady S.A., El-Gawad H.G.A., Ibrahim M.F., Mukherjee S., Elkelish A., Azab E., Gobouri A.A., Farag R., Ibrahim H.A., El-Azm N.A. (2021). Hydrogen peroxide supplementation in irrigation water alleviates drought stress and boosts growth and productivity of potato plants. Sustainability.

[10] Hoque T.S., Hossain M.A., Mostofa M.G., Burritt D.J., Fujita M., Tran L.-S.P. (2016). Methylglyoxal: An emerging signaling molecule in plant abiotic stress responses and tolerance. Front. Plant Sci..

[11] Valderrama R., Corpas F.J., Carreras A., Fernández-Ocaña A., Chaki M., Luque F., Gómez-Rodríguez M.V., Colmenero-Varea P., Luis A., Barroso J.B. (2007). Nitrosative stress in plants. FEBS Lett..

[12] Yadav S.K., Singla-Pareek S.L., Ray M., Reddy M., Sopory S. (2005). Methylglyoxal levels in plants under salinity stress are dependent on glyoxalase I and glutathione. Biochem. Biophys. Res. Commun..

[13] Shohan M.U.S., Sinha S., Nabila F.H., Dastidar S.G., Seraj Z.I. (2019). HKT1;5 transporter gene expression and association of amino acid substitutions with salt tolerance across rice genotypes. Front. Plant Sci..

[14] Prasad P., Djanaguiraman M., Stewart Z., Ciampitti I., Hatfield J.L., Sivakumar M.V.K., Prueger J.H. (2019). Agroclimatology of Maize, Sorghum, and Pearl Millet. Agroclimatology: Linking Agriculture to Climate.

[15] Anglani C. (1998). Sorghum for human food—A review. Plant Foods Hum. Nutr..

[16] Ayub M., Nadeem M.A., Tanveer A., Husnain A. (2002). Effect of different levels of nitrogen and harvesting times on the growth, yield and quality of sorghum fodder. Asian J. Plant Sci..

[17] Joardar J., Razir S., Islam M., Kobir M. (2018). Salinity impacts on experimental fodder sorghum production. SAARC J. Agric..

[18] Abdel-Ghany S.E., Ullah F., Ben-Hur A., Reddy A.S. (2020). Transcriptome analysis of drought-resistant and drought-sensitive sorghum (*Sorghum bicolor*) genotypes in response to PEG-induced drought stress. Int. J. Mol. Sci..

[19] Yan K., Chen P., Shao H., Zhao S., Zhang L., Zhang L., Xu G., Sun J. (2012). Responses of photosynthesis and photosystem II to higher temperature and salt stress in Sorghum. J. Agron. Crop. Sci..

[20] Terzi R., Saruhan G.N., Güven F.G., Kadioglu A. (2018). Alpha lipoic acid treatment induces the antioxidant system and ameliorates lipid peroxidation in maize seedlings under osmotic stress. Arch. Biol. Sci..

[21] Packer L., Witt E.H., Tritschler H.J. (1995). Alpha-lipoic acid as a biological antioxidant. Free Radic. Biol. Med..

[22] Sgherri C., Quartacci M.F., Izzo R., Navari-Izzo F. (2002). Relation between lipoic acid and cell redox status in wheat grown in excess copper. Plant Physiol. Biochem..

[23] Javeed H.M.R., Ali M., Skalicky M., Nawaz F., Qamar R., Faheem M., Mubeen M., Iqbal M.M., Vachova P., Brestic M. (2021). Lipoic Acid Combined with Melatonin Mitigates Oxidative Stress and Promotes Root Formation and Growth in Salt-Stressed Canola Seedlings (*Brassica napus* L.). Molecules.

[24] Sezgin A., Altuntaş C., Demiralay M., Cinemre S., Terzi R. (2019). Exogenous alpha lipoic acid can stimulate photosystem II activity and the gene expressions of carbon fixation and chlorophyll metabolism enzymes in maize seedlings under drought. J. Plant Physiol..

[25] Turk H., Erdal S., Karayel U., Dumlupinar R. (2018). Attenuation of lead toxicity by promotion of tolerance mechanism in wheat roots by lipoic acid. Cereal Res. Commun..

[26] Singh A., Kumar J., Kumar P. (2008). Effects of plant growth regulators and sucrose on post harvest physiology, membrane stability and vase life of cut spikes of gladiolus. Plant Growth Regul..

[27] Abd El-Gawad H.G., Mukherjee S., Farag R., Abd Elbar O.H., Hikal M., Abou El-Yazied A., Abd Elhady S.A., Helal N., ElKelish A., El Nahhas N. (2021). Exogenous γ-aminobutyric acid (GABA)-induced signaling events and field performance associated with mitigation of drought stress in *Phaseolus vulgaris* L.. Plant Signal. Behav..

[28] Velikova V., Yordanov I., Edreva A. (2000). Oxidative stress and some antioxidant systems in acid rain-treated bean plants: Protective role of exogenous polyamines. Plant Sci..

[29] Heath R.L., Packer L. (1968). Photoperoxidation in isolated chloroplasts: I. Kinetics and stoichiometry of fatty acid peroxidation. Arch. Biochem. Biophys..

[30] Costache M.A., Campeanu G., Neata G. (2012). Studies concerning the extraction of chlorophyll and total carotenoids from vegetables. Rom. Biotechnol. Lett..

[31] De Carvalho L.M.J., Gomes P.B., de Oliveira Godoy R.L., Pacheco S., do Monte P.H.F., de Carvalho J.L.V., Nutti M.R., Neves A.C.L., Vieira A.C.R.A., Ramos S.R.R. (2012). Total carotenoid content, α-carotene and β-carotene, of landrace pumpkins (*Cucurbita moschata* Duch): A preliminary study. Food Res. Int..

[32] Bradford M.M. (1976). A rapid and sensitive method for the quantitation of microgram quantities of protein utilizing the principle of protein-dye binding. Anal. Biochem..

[33] Beyer W.F., Fridovich I. (1987). Assaying for superoxide dismutase activity: Some large consequences of minor changes in conditions. Anal. Biochem..

[34] Cakmak I., Strbac D., Marschner H. (1993). Activities of hydrogen peroxide-scavenging enzymes in germinating wheat seeds. J. Exp. Bot..

[35] Dias M.A., Costa M.M. (1983). Effect of low salt concentrations on nitrate reductase and peroxidase of sugar beet leaves. J. Exp. Bot..

[36] Nakano Y., Asada K. (1981). Hydrogen peroxide is scavenged by ascorbate-specific peroxidase in spinach chloroplasts. Plant Cell Physiol..

[37] Havre G.N. (1961). The flame photometric determination of sodium, potassium and calcium in plant extracts with special reference to interference effects. Anal. Chim. Acta.

[38] Livak K.J., Schmittgen T.D. (2001). Analysis of Relative Gene Expression Data Using Real-Time Quantitative PCR and the 2^−ΔΔCT^ Method. Methods.

[39] SAS (1988). SAS/STAT User’s Guide.

[40] Kamal K.Y., Khodaeiaminjan M., Yahya G., El-Tantawy A.A., Abdel El-Moneim D., El-Esawi M.A., Abd-Elaziz M.A., Nassrallah A.A. (2021). Modulation of cell cycle progression and chromatin dynamic as tolerance mechanisms to salinity and drought stress in maize. Physiol. Plant..

[41] Netondo G.W., Onyango J.C., Beck E. (2004). Sorghum and salinity: I. Response of growth, water relations, and ion accumulation to NaCl salinity. Crop Sci..

[42] Skaggs T.H., van Genuchten M.T., Shouse P.J., Poss J.A. (2006). Macroscopic approaches to root water uptake as a function of water and salinity stress. Agric. Water Manag..

[43] Navari-Izzo F., Quartacci M.F., Sgherri C. (2002). Lipoic acid: A unique antioxidant in the detoxification of activated oxygen species. Plant Physiol. Biochem..

[44] Hasan M., Alabdallah N.M., Alharbi B.M., Waseem M., Yao G., Liu X.-D., El-Gawad A., Hany G., El-Yazied A.A., Ibrahim M.F. (2021). GABA: A Key Player in Drought Stress Resistance in Plants. Int. J. Mol. Sci..

[45] Ibrahim M., Ibrahim H.A., Abd El-Gawad H. (2021). Folic acid as a protective agent in snap bean plants under water deficit conditions. J. Hortic. Sci. Biotechnol..

[46] Ibrahim M.F., El-Samad A., Ashour H., El-Sawy A.M., Hikal M., Elkelish A., El-Gawad H.A., El-Yazied A.A., Hozzein W.N., Farag R. (2020). Regulation of agronomic traits, nutrient uptake, osmolytes and antioxidants of maize as influenced by exogenous potassium silicate under deficit irrigation and semiarid conditions. Agronomy.

[47] Jahan M.S., Guo S., Sun J., Shu S., Wang Y., Abou El-Yazied A., Alabdallah N.M., Hikal M., Mohamed M.H., Ibrahim M.F. (2021). Melatonin-mediated photosynthetic performance of tomato seedlings under high-temperature stress. Plant Physiol. Biochem..

[48] Anjum N.A., Sofo A., Scopa A., Roychoudhury A., Gill S.S., Iqbal M., Lukatkin A.S., Pereira E., Duarte A.C., Ahmad I. (2015). Lipids and proteins—Major targets of oxidative modifications in abiotic stressed plants. Environ. Sci. Pollut. Res..

[49] Melkozernov A.N., Blankenship R.E. (2006). Photosynthetic functions of chlorophylls. Chlorophylls and Bacteriochlorophylls.

[50] Parry A.D., Horgan R. (1991). Carotenoids and abscisic acid (ABA) biosynthesis in higher plants. Physiol. Plant..

[51] Seo M., Koshiba T. (2002). Complex regulation of ABA biosynthesis in plants. Trends Plant Sci..

[52] Hasan M., Rahman M.A., Skalicky M., Alabdallah N.M., Waseem M., Jahan M.S., Ahammed G.J., El-Mogy M.M., El-Yazied A.A., Ibrahim M.F. (2021). Ozone Induced Stomatal Regulations, MAPK and Phytohormone Signaling in Plants. Int. J. Mol. Sci..

[53] Ibrahim M. (2014). Induced drought resistance in common bean (*Phaseolus vulgaris* L.) by exogenous application with active yeast suspension. Middle East J. Appl. Sci..

[54] Ibrahim M.F., Elbar O.H.A., Farag R., Hikal M., El-Kelish A., El-Yazied A.A., Alkahtani J., El-Gawad H.G.A. (2020). Melatonin counteracts drought induced oxidative damage and stimulates growth, productivity and fruit quality properties of tomato plants. Plants.

[55] Jing X., Hou P., Lu Y., Deng S., Li N., Zhao R., Sun J., Wang Y., Han Y., Lang T. (2015). Overexpression of copper/zinc superoxide dismutase from mangrove Kandelia candel in tobacco enhances salinity tolerance by the reduction of reactive oxygen species in chloroplast. Front. Plant Sci..

[56] Data P. (1995). Alpha-lipoic acid. Arzneimittelforschung.

[57] Blumwald E. (2000). Sodium transport and salt tolerance in plants. Curr. Opin. Cell Biol..

[58] Gorcek Z., Erdal S. (2015). Lipoic acid mitigates oxidative stress and recovers metabolic distortions in salt-stressed wheat seedlings by modulating ion homeostasis, the osmo-regulator level and antioxidant system. J. Sci. Food Agric..

[59] Elkelish A., El-Mogy M.M., Niedbała G., Piekutowska M., Atia M.A.M., Hamada M.M.A., Shahin M., Mukherjee S., El-Yazied A.A., Shebl M. (2021). Roles of Exogenous α-Lipoic Acid and Cysteine in Mitigation of Drought Stress and Restoration of Grain Quality in Wheat. Plants.

